# Supplementary material for: High-throughput micro-scale bandgap mapping for perovskite-inspired materials with complex composition space
Source: Nat Commun. 2025 Aug 12;16:7477. doi: 10.1038/s41467-025-62774-y (PMC12343849; doi:10.1038/s41467-025-62774-y)
Supplement: Supplementary file 1 — Supplementary information [file 41467_2025_62774_MOESM1_ESM.pdf]

Table of Contents

Supplementary Note 1 Droplet-to-droplet consistency in batch printing

Supplementary Note 2 Round-to-round consistency in gradient printing

Supplementary Note 3 Halide segregation in mixed compositions

Supplementary Note 4 Bandgap extraction algorithm

Supplementary Note 5 Workflow throughput and the total extracted bandgap data points

Supplementary Note 6 Discussions on sample heterogeneity

Supplementary Table 1 Throughput comparison of different characterization methods

Supplementary Table 2 Differences in bandgap extraction algorithms compared to Ref 17

Supplementary Figure 1 Throughput comparison of different characterization methods

Supplementary Figure 2 Comparison of bandgap extraction results between previous work and current manuscript

Supplementary Figure 3 Bandgap statistics of batch-printed samples via spatially-resolved hyperspectral mapping

Supplementary Figure 4 Bandgap distribution histograms of 5 droplets in 9 batch-printed samples

Supplementary Figure 5 Comparison of bandgap distributions between different rounds of gradient printing

Supplementary Figure 6 Bandgap distribution histograms of 5 droplets with the same estimated compositions in three rounds of gradient printing for each sequence

Supplementary Figure 7 XRD patterns of batch-printed droplets

Supplementary Figure 8 XRD and EDS results on gradient-printed $(Cs_3Sb_2I_9)_{1-x}(Cs_3Bi_2Br_9)_x$ sequence

Supplementary Figure 9 PXRD and XRD results on sequence $(Cs_3Sb_2I_9)_{1-x}(Cs_3Bi_2Br_9)_x$

Supplementary Figure 10 PXRD and bandgap analysis on halide segregation in mixed composition $(Cs_3Sb_2Br_9)_{0.5}(Cs_3Bi_2I_9)_{0.5}$

Supplementary Figure 11 SEM-EDS mapping on drop-casted sample with the composition $(Cs_3Sb_2Br_9)_{0.5}(Cs_3Bi_2I_9)_{0.5}$

Supplementary Figure 12 Bandgap of six perovskite material gradients

Supplementary Figure 13 Spatially resolved bandgap analysis results and histograms of all droplets in sequence $(Cs_3Br_2I_9)_{1-x}(Cs_3Bi_2Br_9)_x$ from 6 rounds of experiments

Supplementary Figure 14 Example local bandgap maps of droplets from the composition gradient sequence $(Cs_3Br_2I_9)_{1-x}(Cs_3Bi_2Br_9)_x$

Supplementary Figure 15 Histograms of the bandgap distribution for droplets with different compositions

Supplementary Figure 16 Local bandgap maps of spin-coated thin films with compositions corresponding to batch-printed samples

Supplementary Figure 17 Two-dimensional TA spectra and TA spectra at different time delays of four pure compositions

Supplementary Figure 18 TA spectra of four pure compositions in three rounds of scan

Supplementary Figure 19 Normalized transient absorption kinetics at two GSB peak positions

Supplementary Figure 20 Examples of lifetime fitting in transient absorption kinetics

Supplementary Figure 21 Bandgap comparison between gradient-printed samples and spin-coated films

Supplementary Figure 22 Local bandgap maps of drop-casted $Cs_3(Bi_{0.5}Sb_{0.5})_2(Br_{0.5}I_{0.5})_9$ droplet and spin-coated $MA_{0.5}FA_{0.5}Pb(I_{0.5}Br_{0.5})_3$ film before and after 10-minute illumination

Supplementary Table

Supplementary Table 1. Throughput comparison of different characterization methods

Method	Acquisition Time/ Measurement	Damage to Sample	Accessibility	Ref
XRD	5-60 mins	moderate	Widely available	[5]
UV-Vis	1-5 mins	low	Widely available	empirical
SEM	2-10 mins	moderate	Widely available	empirical
High-throughput GIWAXS	10-60 s	moderate	Limited access	[7]
High-throughput PL	5-10 s	moderate	customized	[6]
High-throughput EDX	80-105 s	moderate	customized	[35]
High-throughput bandgap	10-20 μs	low	customized	This work

Supplementary Table 2. Differences in bandgap extraction algorithms compared to Ref 17

	Our work	Previous work[17]
Range	Full data range	Manual selection of target bandgap range
Resolution	Spatially-resolved bandgaps (N x N spectra)	Average bandgap of each droplet (1 spectrum)
Fitting	Linear regression on maximum difference	Linear regression on detected peaks

Supplementary Note

Supplementary Note 1. Droplet-to-droplet consistency in batch printing

All droplets were deposited with identical motor pumping rates, precursors, and substrate temperatures. However, uncontrollable factors may lead to printing variations. For example, air bubbles generated in plumbing lines during printing could result in differences in droplet sizes, shapes and thicknesses. To evaluate the droplet-to-droplet consistency in one sample from batch printing, we performed hyperspectral measurement and spatial-resolved bandgap analysis on nine samples. As shown in **Supplementary Figure 3**, all nine pictures have a horizontal band, indicating that the bandgap distribution ranges are consistent across all droplets. In addition, the dark area and light area are well aligned between droplets. It is obvious that droplets capture two peaks in bandgap distribution in mixture $Cs_3(Bi_{0.5}Sb_{0.5})_2(Br_{0.5}I_{0.5})_9$. With the high consistency, we can eliminate the concern that the differences in droplet morphology can greatly influence the bandgap.

Supplementary Note 2. Round-to-round consistency in gradient printing

Constrained by our parameter settings, the gradient printing can only print 75% of the gradient sequence on one glass slide (from pure A to $A_{0.25}B_{0.75}$). Therefore, we combine one forward printing and one backward printing, overlapping in the compositional range of 25-75%, and refer to it as a single round of print. The consistency can be assessed by the overlapping area and by comparing the bandgap distribution of each round. The bandgap distribution of each round in six sequences is highlighted in pink, as depicted in **Supplementary Figure 5**. For better visualization, we also plotted histograms of the bandgap distributions for the same compositions in the gradient for each round (**Supplementary Figure 6**). We find no discernible offset in the composition region of 25-75%, and the distribution pattern between each round exhibits high similarity.

Supplementary Note 3. Halide segregation in mixed compositions

As shown in **Supplementary Fig. 9a**, the pattern of the composition $(Cs_3Sb_2I_9)_{0.75}(Cs_3Bi_2Br_9)_{0.25}$, located in the 0D-2D structural transition region, implies the coexistence of 0D and 2D structures, with the peaks at 26.2º and 30.5º corresponding to the 0D structure and the peak at 27.8º corresponding to the 2D structure. The slight deviations from the reference patterns result from lattice changes after mixing B-site and X-site ions. For the composition $(Cs_3Sb_2I_9)_{0.5}(Cs_3Bi_2Br_9)_{0.5}$, the pattern demonstrates a 2D structure when compared to the reference pattern $Cs_3Bi_2Br_9$, with all peaks shifting to the left. Additionally, the peaks are broadened and asymmetric (also shown in **Supplementary Fig. 10**), indicating elemental segregation. However, it is challenging to identify the exact phases that share the same 2D structure but possess slightly different compositions. For the composition $(Cs_3Sb_2I_9)_{0.75}(Cs_3Bi_2Br_9)_{0.25}$, which just exceeds the mixed-phase region, the main peaks become thinner compared to $(Cs_3Sb_2I_9)_{0.5}(Cs_3Bi_2Br_9)_{0.5}$. The results of thin-film XRD generally show broader peaks compared to pXRD due to lower crystallinity, making it difficult to detect elemental segregation by peak width. However, it is easier to identify 0D-2D structural changes with characteristic patterns. Clearly, $Cs_3Bi_2Br_9$, $(Cs_3Sb_2I_9)_{0.75}(Cs_3Bi_2Br_9)_{0.25}$, and $(Cs_3Sb_2I_9)_{0.5}(Cs_3Bi_2Br_9)_{0.5}$ exhibit similar diffraction patterns with a gradual left shift, indicating that all of them have a 2D $Cs_3Bi_2Br_9$ structure. However, $(Cs_3Sb_2I_9)_{0.75}(Cs_3Bi_2Br_9)_{0.25}$ shows a mixture of both 0D and 2D characteristics, aligning with the conclusion that the structural transition occurs in the range of x = [0.10, 0.30] in the sequence $(Cs_3Sb_2I_9)_x(Cs_3Bi_2Br_9)_{1-x}$. We noticed that peak broadening occurred in mixed compositions when conducting XRD measurements on batch-printed samples (**Supplementary Figure 10**), especially in $(Cs_3Sb_2I_9)_{0.5}(Cs_3Bi_2Br_9)_{0.5}$. This indicated potential phase segregation happened in printed samples which caused the wide distribution in bandgaps. To dive deep into this phenomenon, we chose composition $Cs_3(Bi_{0.5}Sb_{0.5})_2(I_{0.5}Br_{0.5})_9$ and performed powder X-ray diffraction (PXRD). Since powder scratched from multiple spin-coated films was still too limited for high-quality PXRD, we drop-casted

precursor solution on multiple glass substrates and annealed until all solvents were baked out. Then the material was scratched from those substrates (to achieve the amount for analysis) and ground into powder for measurement. By comparing the PXRD pattern with reference patterns, we observed that the peaks in the experimental pattern matched well with the 2D $Cs_3Bi_2Br_9$ reference, with a slight peak shift to lower two-theta angles, implying that the mixed composition $Cs_3(Bi_{0.5}Sb_{0.5})_2(Br_{0.5}I_{0.5})_9$ has a purely 2D phase (**Supplementary Figure 10**). Additionally, peak broadening and asymmetry were observed even in the powdered sample, which could result from either mixed 2D and 0D phases or elemental segregation. Given the 2D structure of $Cs_3(Bi_{0.5}Sb_{0.5})_2(Br_{0.5}I_{0.5})_9$, the peak broadening and asymmetry are most likely due to elemental segregation. This conclusion is further supported by the varying bandgap observed in optical measurements. We further fitted the broadened peak with several Gaussian peaks with identical widths but flexible intensities. We chose two-peak fitting instead of multi-peak fitting as this was more consistent with observations from optical bandgap analysis and transient absorption. However, this is a rough calculation, and the extra small peaks in optical bandgap distribution could be accounted for the residuals in two-peak fitting, suggesting that the halide segregation has continuous variation in halide proportion across the sample. To further support the XRD results, EDX mapping (**Supplementary Figure 11**) was performed on drop-casted samples with the composition $Cs_3(Bi_{0.5}Sb_{0.5})_2(Br_{0.5}I_{0.5})_9$. The distribution of elements are non-uniform with the droplet, indicating elemental segregation.

Supplementary Note 4. Bandgap extraction algorithm

For automatic bandgap extraction, we modified the published Autocharaterization algorithm[32]. We improved the accuracy and robustness of the fitting algorithm and added pixel-wise bandgap calculation. In vision.py, we output the X and Y coordinates of each pixel after droplet segmentation. In

bandextractor.py, we input the whole range of the reflectance spectrum. After running recursive segmentation, the difference of each segmented line was calculated (see Python code below). The linear fitting model was applied to the segmented line with the largest difference value. Finally, the bandgap was determined by the intersection between the fitting line and the background baseline.

```
#### input ####
# - num_drop:         Number of droplets extracted from the segmentation algorithm
# - csv_path:         Data path to the raw input CSV file with an array dimension of (n, m+1), where n represents the wavelength channels
and m is the number of pixels within one droplet. The first column comprises a list of wavelengths, while the other columns contain
reflectance spectra (in decimal) of individual pixels.
############

for idx in range(num_drop):
    # Read data from the CSV file
    data = pd.read_csv(csv_path)

    # Extract relevant columns from the data
    R = data.iloc[:, 2:]  # Extracting reflectance array for Tauc plot
    wl = data.iloc[:, 1]     # Extracting wavelength data

    # Calculate k and s values for absorption coefficient calculation
    k = (1. - R) ** 2  # k=(1-R)^2
    s = 2 * R  # s = 2*R
    F = k / s  # Absorpotion coefficient
    ev = 1240. / wl  # Calcualte eV from wavelength
    ev.name = 'eV'  # Rename column
    tauc = F.mul(ev, axis=0) ** 2.  # Calculate tauc
    tauc = pd.concat([ev, tauc], axis=1)  # Add eV column back to data

    # Perform Savitzky-Golay smoothing on Tauc data
    tauc_smooth_raw = tauc.copy()
    smooth = scipy.signal.savgol_filter(tauc.iloc[:, 1:], window_length=20, polyorder=3, axis=0)

    # Upsample the data points from ~100 to 1000
    upsample = 1000  # number of points to upsample to
    f = scipy.interpolate.interp1d(tauc_smooth_raw.iloc[:, 0], smooth, axis=0)
    ev_upsample = np.linspace(np.max(tauc_smooth_raw.iloc[:, 0]),
                              np.min(tauc_smooth_raw.iloc[:, 0]), upsample)
    tauc_smooth_1 = pd.DataFrame(np.hstack([ev_upsample.reshape(upsample, 1), f(ev_upsample)]),
                                columns=tauc_smooth_raw.columns.values)
    tauc_smooth = tauc_smooth_1.iloc[::-1].reset_index(drop=True)  # sort ascending eV

    # Initialize lists for bandgaps and absorption maxima
    bandgaps = []
    absorption_max = []
    for i in range(tauc_smooth.shape[1] - 1):
        bandgaps_per_tauc = []
        current_tauc = tauc_smooth.iloc[:, i + 1].name  # name of current tauc spectra
        TAUC_X = np.array(tauc_smooth.iloc[:, 0]).reshape(-1, 1)
        TAUC_Y = np.array(tauc_smooth.iloc[:, i + 1]).reshape(-1, 1)
        X0 = [TAUC_X]  # initialize X values
        Y0 = [TAUC_Y]  # initialize Y values
        target_len = len(X0[0])  # target length to stop recursion
        R_tol = 0.995  # R^2 linear regression fit tolerance for line segments
```

```
X_tol = []  # X segments above R_tol
Y_tol = []  # Y segments above R_tol
m = []  # list of slopes
current_len = 0

# Perform recursive segmentation
while current_len < target_len:
    X = []
    Y = []
    for segX, segY in zip(X0, Y0):
        mid = len(segX) // 2
        # left segments
        X_L = segX[:mid + 1]  # left segment
        Y_L = segY[:mid + 1]  # left segment
        model_L = LinearRegression().fit(X_L, Y_L)
        if model_L.score(X_L, Y_L) >= R_tol:
            X_tol.append(X_L)
            Y_tol.append(Y_L)
            m.append(model_L.coef_.item())
        else:
            X.append(X_L)
            Y.append(Y_L)
        # right segments
        X_R = segX[mid:]  # right segment
        Y_R = segY[mid:]  # right segment
        model_R = LinearRegression().fit(X_R, Y_R)
        if model_R.score(X_R, Y_R) >= R_tol:
            X_tol.append(X_R)
            Y_tol.append(Y_R)
            m.append(model_R.coef_.item())
        else:
            X.append(X_R)
            Y.append(Y_R)
    X0 = X  # reinit
    Y0 = Y  # reinit
    # count num of element in X_tol. When X_tol == target_len, end recursion.
    current_len = 0
    medians = []  # Get list of all medians to sort list of lists later
    for l in X_tol:
        current_len += len(l)
        medians.append(np.median(l))

# sort lists of lists based on X_tol order
sort_mask = np.argsort(medians)
X_tol_sort = np.array(X_tol, dtype=object)[sort_mask]
Y_tol_sort = np.array(Y_tol, dtype=object)[sort_mask]


# calcualte inclination angles between segment slopes and x-axis
thetas = np.rad2deg(np.arctan(np.array(m)[sort_mask]))

Delta = []
for k in range(0, len(X_tol_sort)):
    if X_tol_sort[k][0] > 2.70 :
        break
upper = k
for i in range(0, k):
    d = Y_tol_sort[i][len(Y_tol_sort[i])-1] - Y_tol_sort[i][0]
    Delta.append(d)
Delta = np.array(Delta, dtype = float)
i0 = np.argmax(Delta)
delta_max = np.max(Delta)
```

```
        if i0 == 0:
            Eg = 0.
            bandgaps.append(Eg)
            continue

        base_range = Y_tol_sort[0]
        for i in range(1, i0):
            base_range = np.concatenate((base_range, Y_tol_sort[i]))
        base = np.median(base_range)

        model = LinearRegression().fit(np.vstack(X_tol_sort[i0]]),
                                       np.vstack([Y_tol_sort[i0] - base]))
        y_fit = model.predict(TAUC_X)
        tngt = LineString([(np.min(TAUC_X), np.min(y_fit)), (np.max(TAUC_X), np.max(y_fit))])
        xax = LineString([(np.min(TAUC_X), 0.), (np.max(TAUC_X), 0.)])
        int_pt = tngt.intersection(xax)
        if hasattr(int_pt, 'x'):
            Eg = int_pt.x  # bandgap, if no x-intercept, will throw an error
            bandgaps.append(Eg)
        else:
            Eg = 0.
            bandgaps.append(Eg)
            Continue

    # Create DataFrame for band gaps and save to a CSV file
    EG = pd.DataFrame(bandgaps).T.set_axis(tauc_smooth.columns.values[1:], axis=1)
    EG = EG.set_index(f'bandgap{n}' for n in range(EG.shape[0]))
    EG = EG.iloc[0,:] # select only first row
    pixel_coordinates = pd.read_csv(r'pixel_coordinates_{idx}.csv', index_col=0)
    pixel_coordinates['bandgap'] = EG.values
    pixel_coordinates.to_csv(r'Extracted_Band_Gaps_{idx}.csv')
```

Supplementary Note 5. Workflow throughput and the total extracted bandgap data points

The total number of bandgap data points collected was nearly 1 million, spanning six experimental rounds with three forward and three backward printing sequences. Each round involved printing approximately 85 droplets on a glass substrate. Although droplet sizes varied, each droplet typically contained 200 to 300 pixels. As shown in **Fig. 2**, N represents the number of bandgap data points for each sequence, with a total of 993,798 data points collected across all six sequences. The throughput of our characterization method is approximately 100,000 bandgap data points per hour, with the process involving hyperspectral camera scanning and spatially-resolved spectra extraction. Assuming each droplet has 200 pixels and that both scanning and extraction take 5 minutes, 102,000 (200 × 85 × 6) bandgaps can be measured and extracted in one hour.

Supplementary Note 6. Discussions on sample heterogeneity. Sample heterogeneity can be influenced by many factors. From a thermodynamic perspective, some mixed compositions are more stable when they decompose into multiple different compositions because the increasing entropy cannot compensate for the increasing enthalpy. Some compositions are inclined to be well-mixed since the mixing entropy enhances phase stability. From a kinetic perspective, the degree of heterogeneity is affected by crystallization conditions such as solvent, annealing temperature, and time. In this case, heterogeneity is primarily driven by the form factor of the droplets. During the drop-casting process, thick droplets require a longer time for solvent evaporation, leading to a slower crystallization process and a higher degree of heterogeneity. This heterogeneity is unavoidable due to the synthesis method, even if we change other parameters like precursor concentration and annealing temperature.

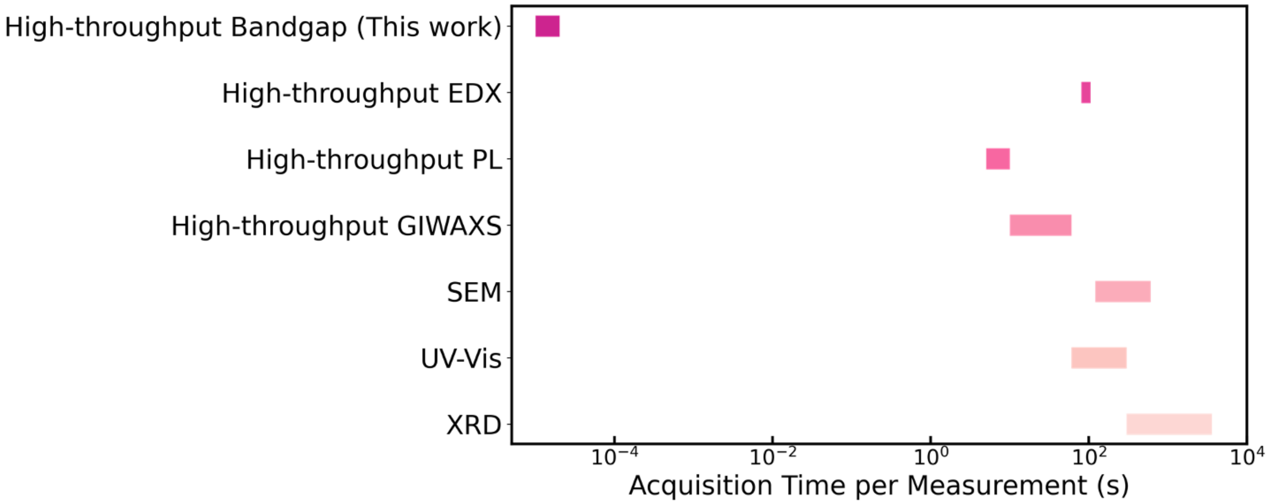

Supplementary Figure 1. Throughput comparison of different characterization methods.

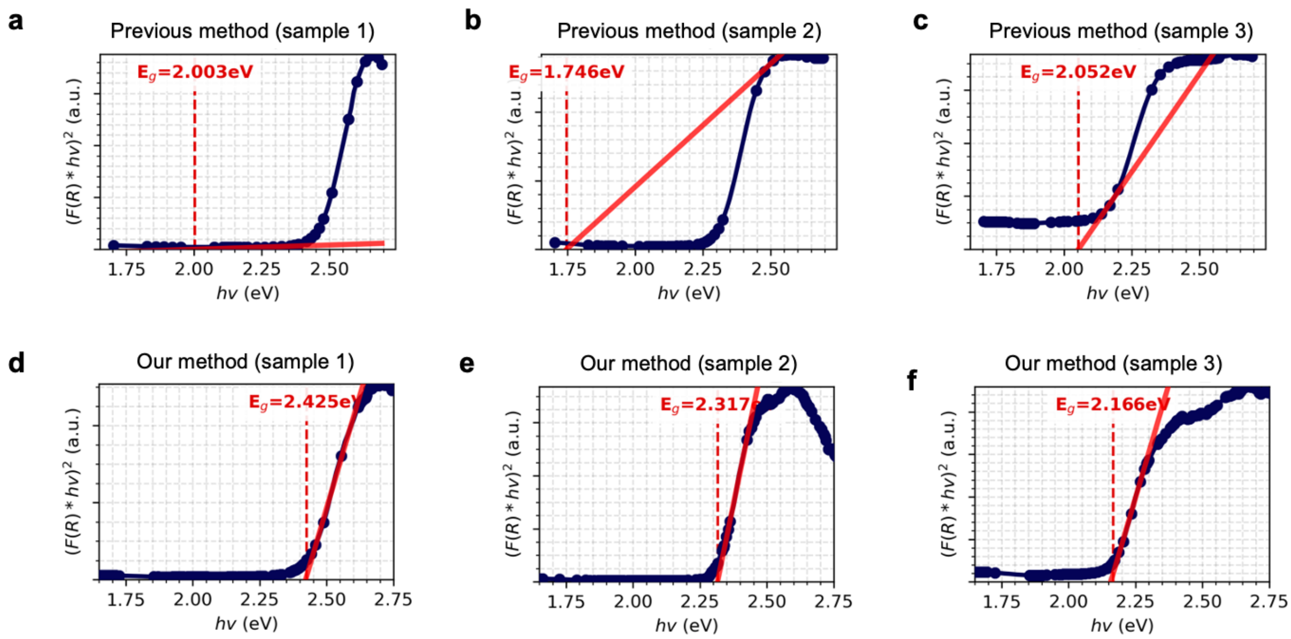

Supplementary Figure 2. Comparison of bandgap extraction results between previous work and current manuscript. a-c, Examples of wrong bandgap extraction results using the algorithm from previous work. **d-f**, Examples of correct bandgap extraction results using the algorithm from this work.

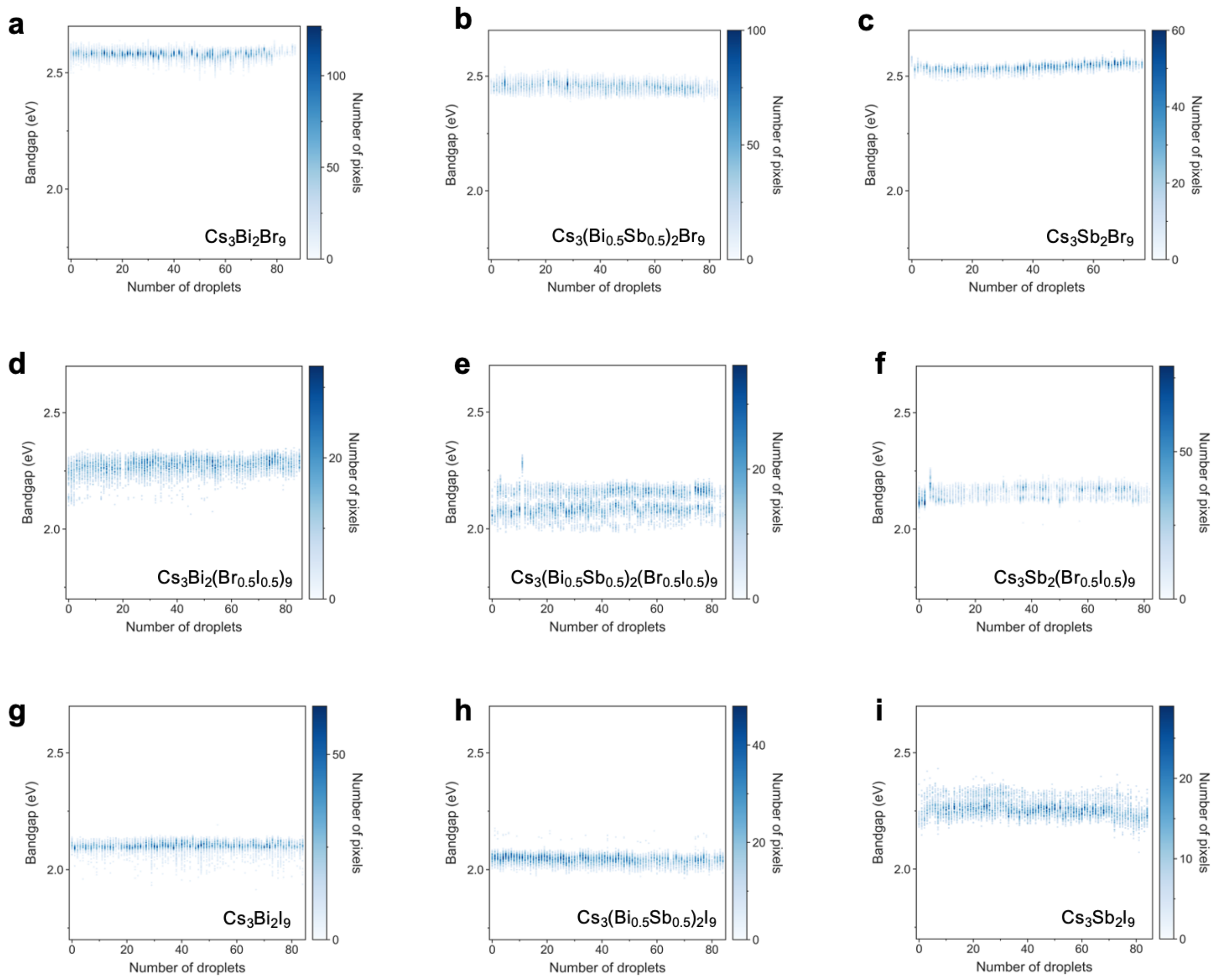

Supplementary Figure 3. Bandgap statistics of batch-printed samples with compositions a, $Cs_3Bi_2Br_9$, **b,** $Cs_3(Bi_{0.5}Sb_{0.5})_2Br_9$, **c,** $Cs_3Sb_2Br_9$, **d,** $Cs_3Bi_2(Br_{0.5}I_{0.5})_9$, **e,** $Cs_3(Bi_{0.5}Sb_{0.5})_2(Br_{0.5}I_{0.5})_9$, **f,** $Cs_3Sb_2(Br_{0.5}I_{0.5})_9$, **g,** $Cs_3Bi_2I_9$, **h,** $Cs_3(Bi_{0.5}Sb_{0.5})_2I_9$, **i,** $Cs_3Sb_2I_9$ **via spatially-resolved hyperspectral mapping.**

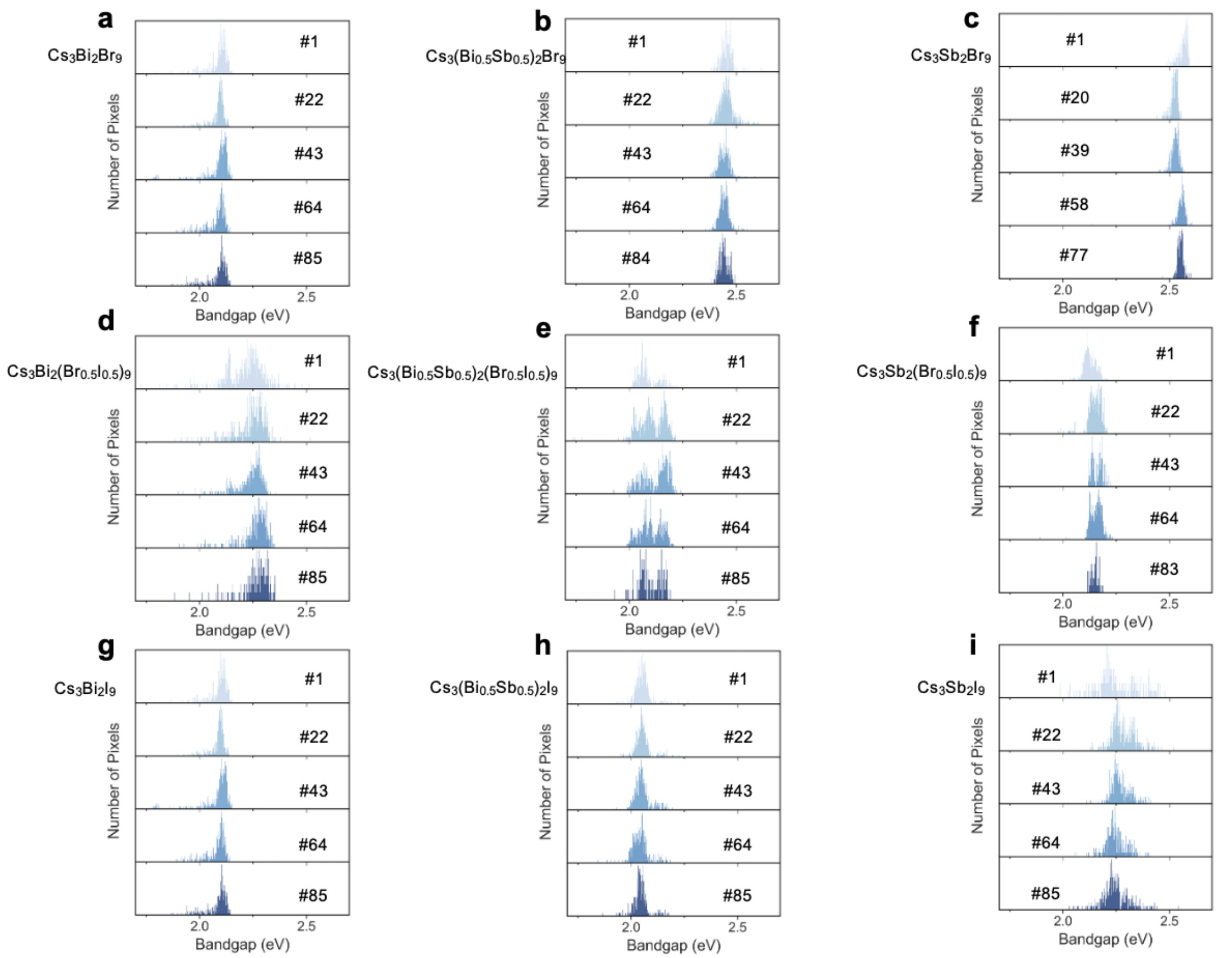

Supplementary Figure 4. Bandgap distribution histograms of 5 droplets in 9 batch-printed samples with compositions a, $Cs_3Bi_2Br_9$, **b,** $Cs_3(Bi_{0.5}Sb_{0.5})_2Br_9$, **c,** $Cs_3Sb_2Br_9$, **d,** $Cs_3Bi_2(Br_{0.5}I_{0.5})_9$, **e,** $Cs_3(Bi_{0.5}Sb_{0.5})_2(Br_{0.5}I_{0.5})_9$, **f,** $Cs_3(Bi_{0.5}Sb_{0.5})_2I_9$, **g,** $Cs_3Bi_2I_9$, **h,** $Cs_3(Bi_{0.5}Sb_{0.5})_2I_9$, **i,** $Cs_3Sb_2I_9$. The number represents the index of droplets in the batch-printed samples.

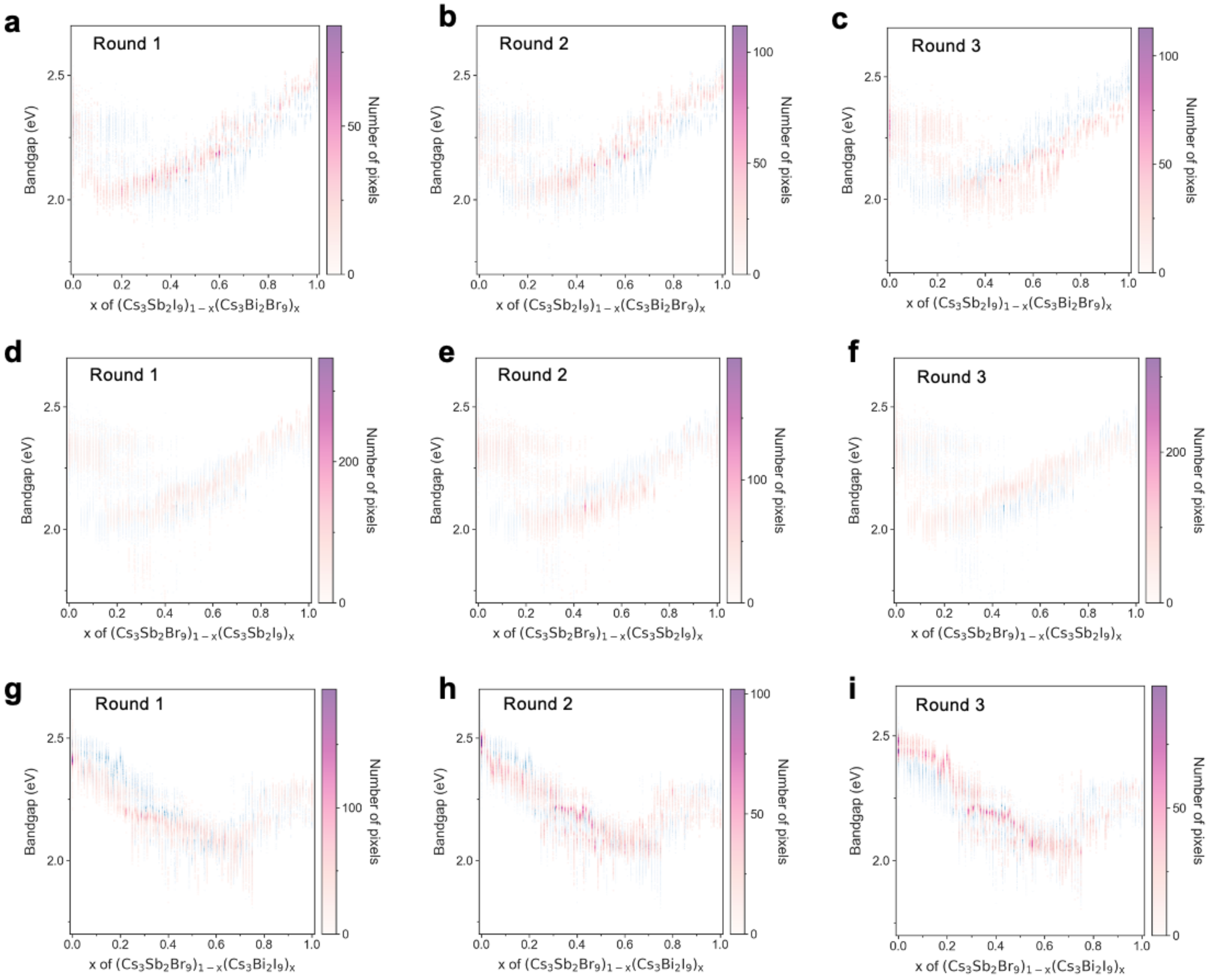

a
Round 1
Bandgap (eV)
Number of pixels
x of $(Cs_3Sb_2I_9)_{1-x}(Cs_3Bi_2Br_9)_x$
b
Round 2
Bandgap (eV)
Number of pixels
x of $(Cs_3Sb_2I_9)_{1-x}(Cs_3Bi_2Br_9)_x$
c
Round 3
Bandgap (eV)
Number of pixels
x of $(Cs_3Sb_2I_9)_{1-x}(Cs_3Bi_2Br_9)_x$
d
Round 1
Bandgap (eV)
Number of pixels
x of $(Cs_3Sb_2Br_9)_{1-x}(Cs_3Sb_2I_9)_x$
e
Round 2
Bandgap (eV)
Number of pixels
x of $(Cs_3Sb_2Br_9)_{1-x}(Cs_3Sb_2I_9)_x$
f
Round 3
Bandgap (eV)
Number of pixels
x of $(Cs_3Sb_2Br_9)_{1-x}(Cs_3Sb_2I_9)_x$
g
Round 1
Bandgap (eV)
Number of pixels
x of $(Cs_3Sb_2Br_9)_{1-x}(Cs_3Bi_2I_9)_x$
h
Round 2
Bandgap (eV)
Number of pixels
x of $(Cs_3Sb_2Br_9)_{1-x}(Cs_3Bi_2I_9)_x$
i
Round 3
Bandgap (eV)
Number of pixels
x of $(Cs_3Sb_2Br_9)_{1-x}(Cs_3Bi_2I_9)_x$

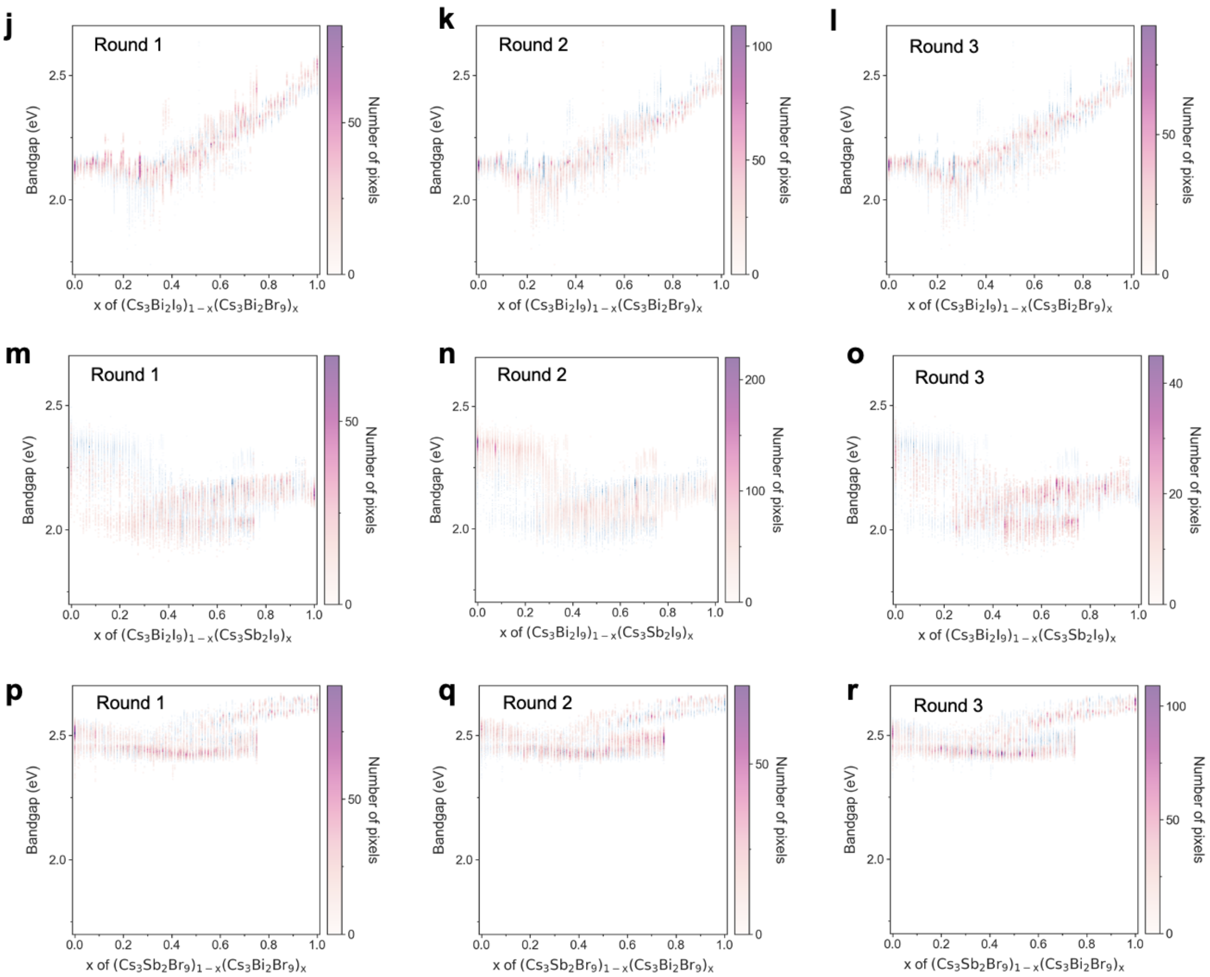

Supplementary Figure 5. Comparison of bandgap distributions between different rounds of gradient printing. The current round was highlighted in pink. The other two rounds are represented in blue. **a-c,** Round 1-3 of sequence $(Cs_3Sb_2I_9)_{1-x}$ $(Cs_3Bi_2Br_9)_x$. **d-f,** Round 1-3 of sequence $(Cs_3Sb_2Br_9)_{1-x}$ $(Cs_3Sb_2I_9)_x$. **g-i,** Round 1-3 of sequence $(Cs_3Sb_2Br_9)_{1-x}$ $(Cs_3Bi_2I_9)_x$. **j-l,** Round 1-3 of sequence $(Cs_3Br_2I_9)_{1-x}$ $(Cs_3Bi_2Br_9)_x$. **m-o,** Round 1-3 of sequence $(Cs_3Bi_2I_9)_{1-x}$ $(Cs_3Sb_2I_9)_x$. **p-r,** Round 1-3 of sequence $(Cs_3Sb_2Br_9)_{1-x}$ $(Cs_3Bi_2Br_9)_x$.

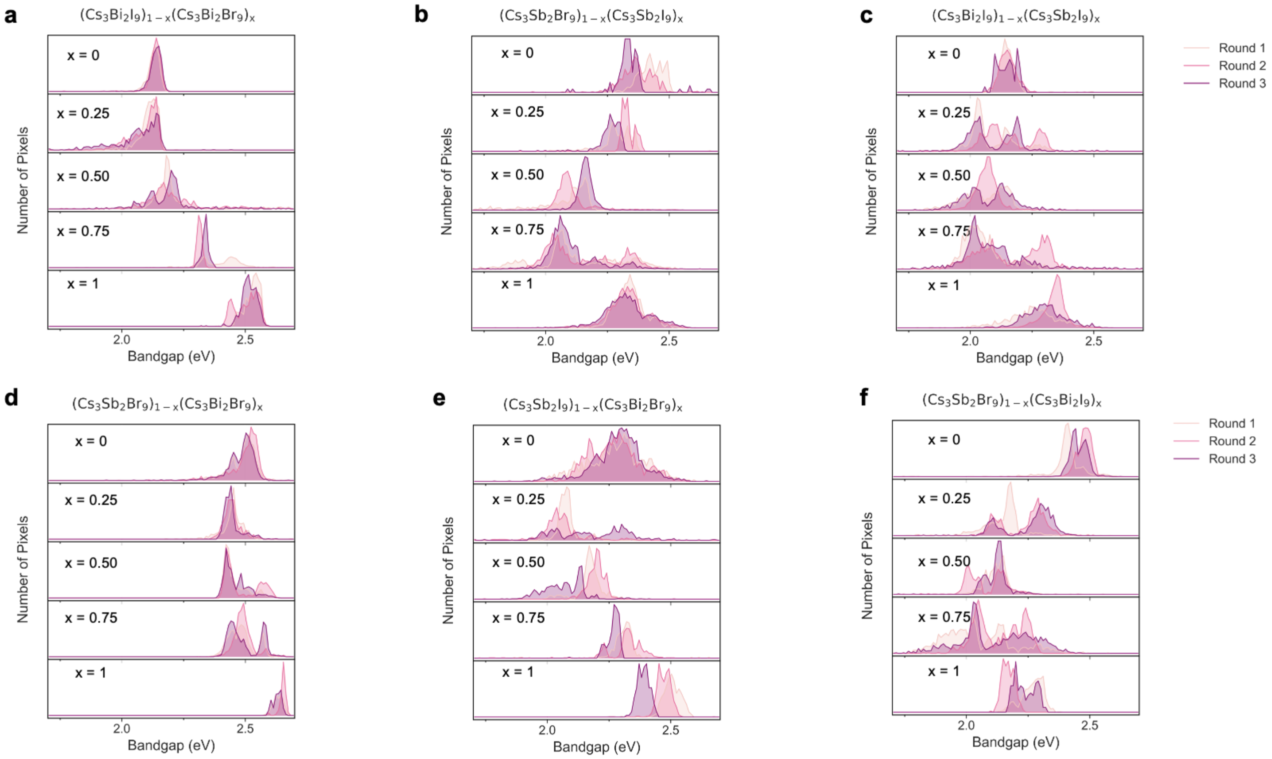

Supplementary Figure 6. Bandgap distribution histograms of 5 droplets with the same estimated compositions in three rounds of gradient printing for each sequence. a, Sequence $(Cs_3Br_2I_9)_{1-x}$ $(Cs_3Bi_2Br_9)_x$. **b**, Sequence $(Cs_3Sb_2Br_9)_{1-x}$ $(Cs_3Sb_2I_9)_x$. **c**, Sequence $(Cs_3Bi_2I_9)_{1-x}$ $(Cs_3Sb_2I_9)_x$. **d**, Sequence $(Cs_3Sb_2Br_9)_{1-x}$ $(Cs_3Bi_2Br_9)_x$. **e**, Sequence $(Cs_3Sb_2I_9)_{1-x}$ $(Cs_3Bi_2Br_9)_x$. **f**, Sequence $(Cs_3Sb_2Br_9)_{1-x}$ $(Cs_3Bi_2I_9)_x$.

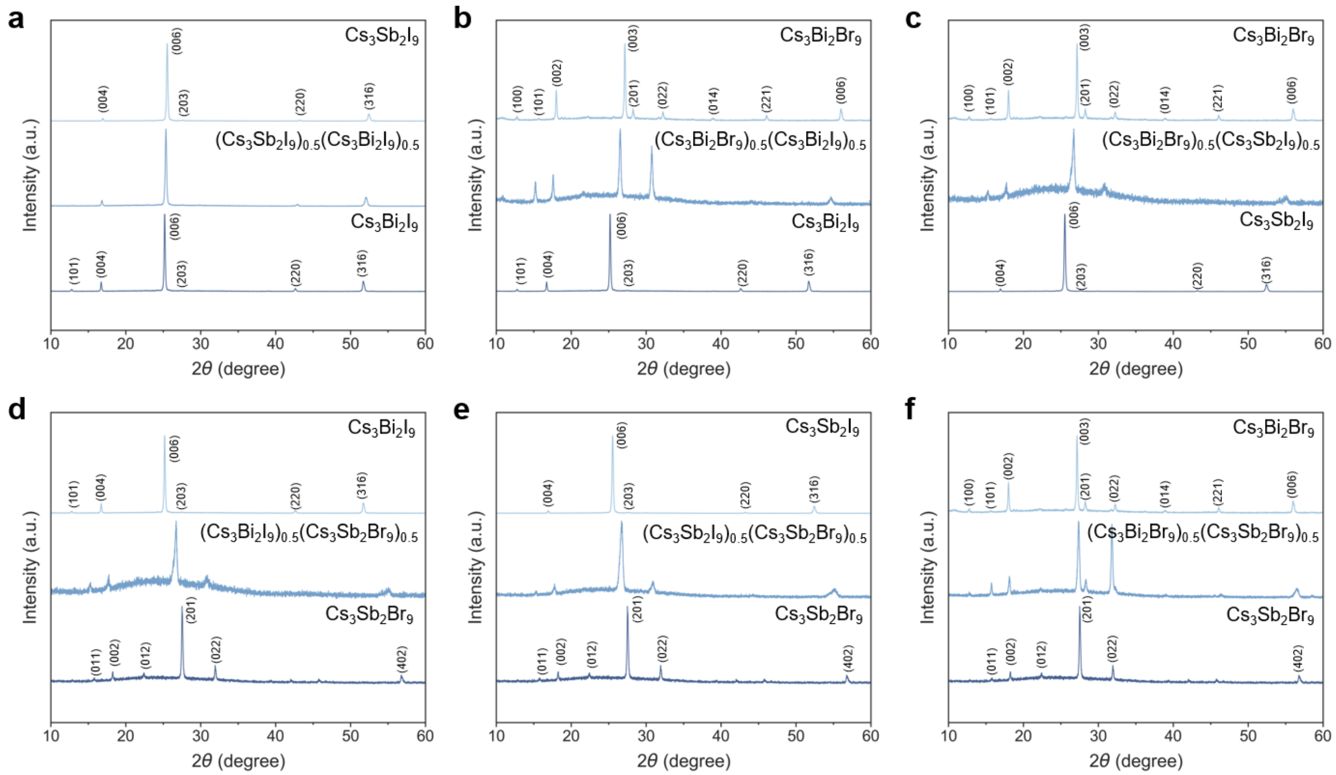

Supplementary Figure 7. XRD patterns of batch-printed droplets. a, XRD patterns of selected compositions from sequence $(Cs_3Bi_2I_9)_{1-x}$ $(Cs_3Sb_2I_9)_x$. “a.u.” denotes “arbitrary units”. **b,** XRD patterns of selected compositions from sequence $(Cs_3Br_2I_9)_{1-x}$ $(Cs_3Bi_2Br_9)_x$. “a.u.” denotes “arbitrary units”. **c,** XRD patterns of selected compositions from sequence $(Cs_3Sb_2I_9)_{1-x}$ $(Cs_3Bi_2Br_9)_x$. “a.u.” denotes “arbitrary units”. **d,** XRD patterns of selected compositions from sequence $(Cs_3Sb_2Br_9)_{1-x}$ $(Cs_3Bi_2I_9)_x$. “a.u.” denotes “arbitrary units”. **e,** XRD patterns of selected compositions from sequence $(Cs_3Sb_2Br_9)_{1-x}$ $(Cs_3Sb_2I_9)_x$. “a.u.” denotes “arbitrary units”. **f,** XRD patterns of selected compositions from sequence $(Cs_3Sb_2Br_9)_{1-x}$ $(Cs_3Bi_2Br_9)_x$. “a.u.” denotes “arbitrary units”.

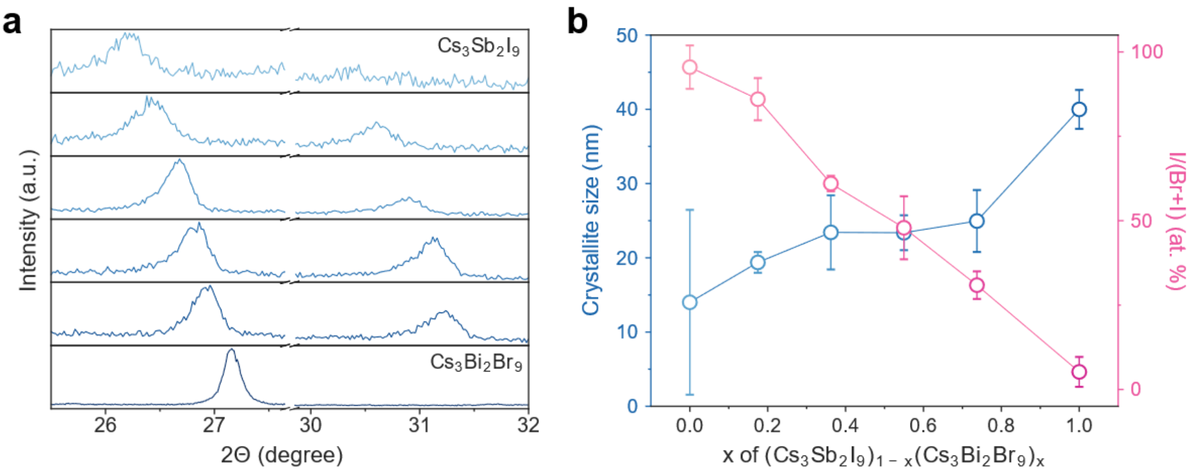

Supplementary Figure 8. XRD and EDS results on gradient-printed $(Cs_3Sb_2I_9)_{1\text{-}x}(Cs_3Bi_2Br_9)_x$ sequence. a, XRD patterns. “a.u.” denotes “arbitrary units”. **b**, Crystallite size (left) and the halide atomic ratios (right) extracted from XRD patterns and SEM-EDS results. The error bar represents standard deviations calculated from two measurements.

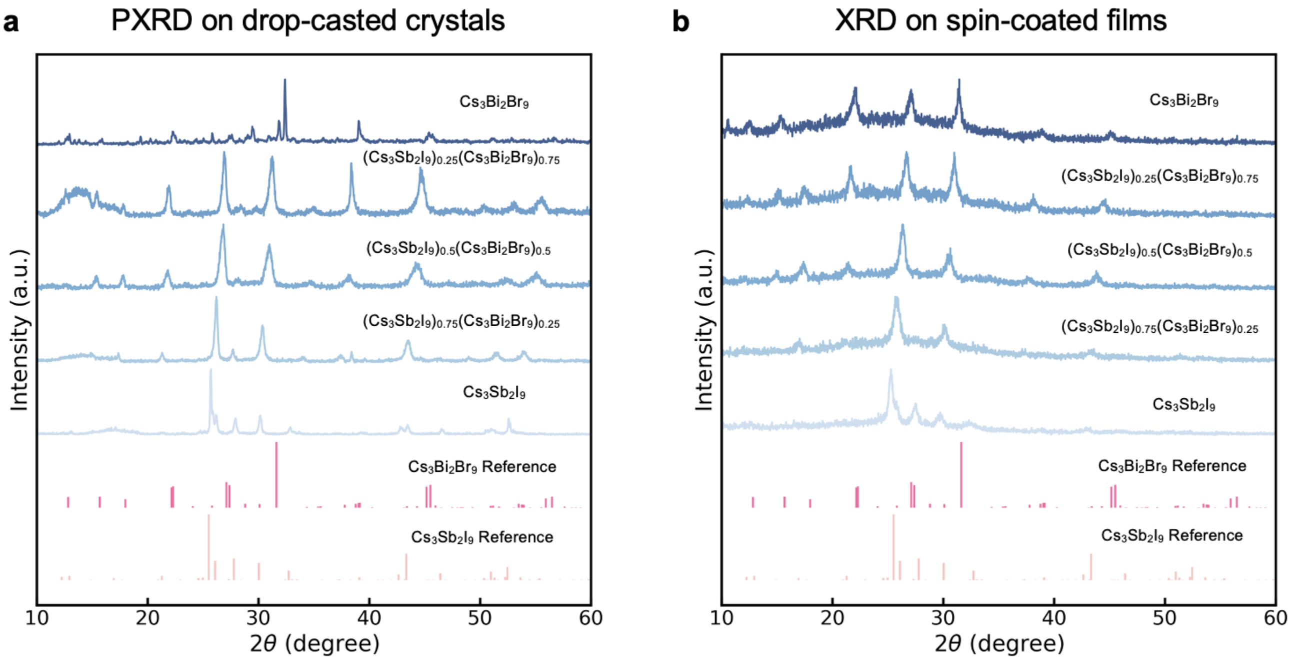

Supplementary Figure 9. PXRD and XRD results on sequence $(Cs_3Sb_2I_9)_{1-x}(Cs_3Bi_2Br_9)_x$. a, PXRD patterns of drop-casted crystals. “a.u.” denotes “arbitrary units”. **b**, XRD patterns of spin-coated films. “a.u.” denotes “arbitrary units”.

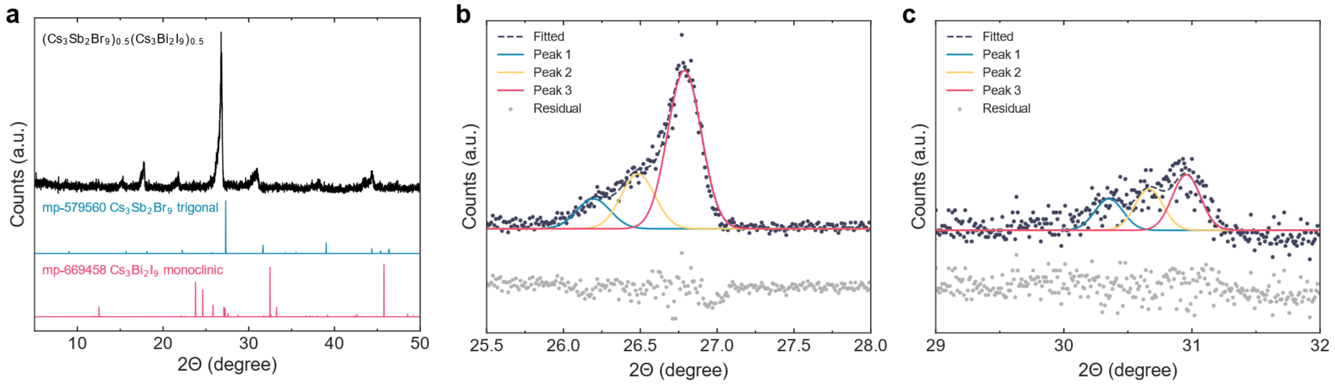

Supplementary Figure 10. Powder XRD (PXRD) and bandgap analysis suggest halide segregation in mixed composition $(Cs_3Sb_2Br_9)_{0.5}(Cs_3Bi_2I_9)_{0.5}$. a, PXRD pattern of $(Cs_3Sb_2Br_9)_{0.5}(Cs_3Bi_2I_9)_{0.5}$ which indicates trigonal phase. “a.u.” denotes “arbitrary units”. **b** and **c,** Three-peak Gaussian fit and the residual plots for broadened peaks at (**b**) 26.8° and (**c**) 30.7°. “a.u.” denotes “arbitrary units”.

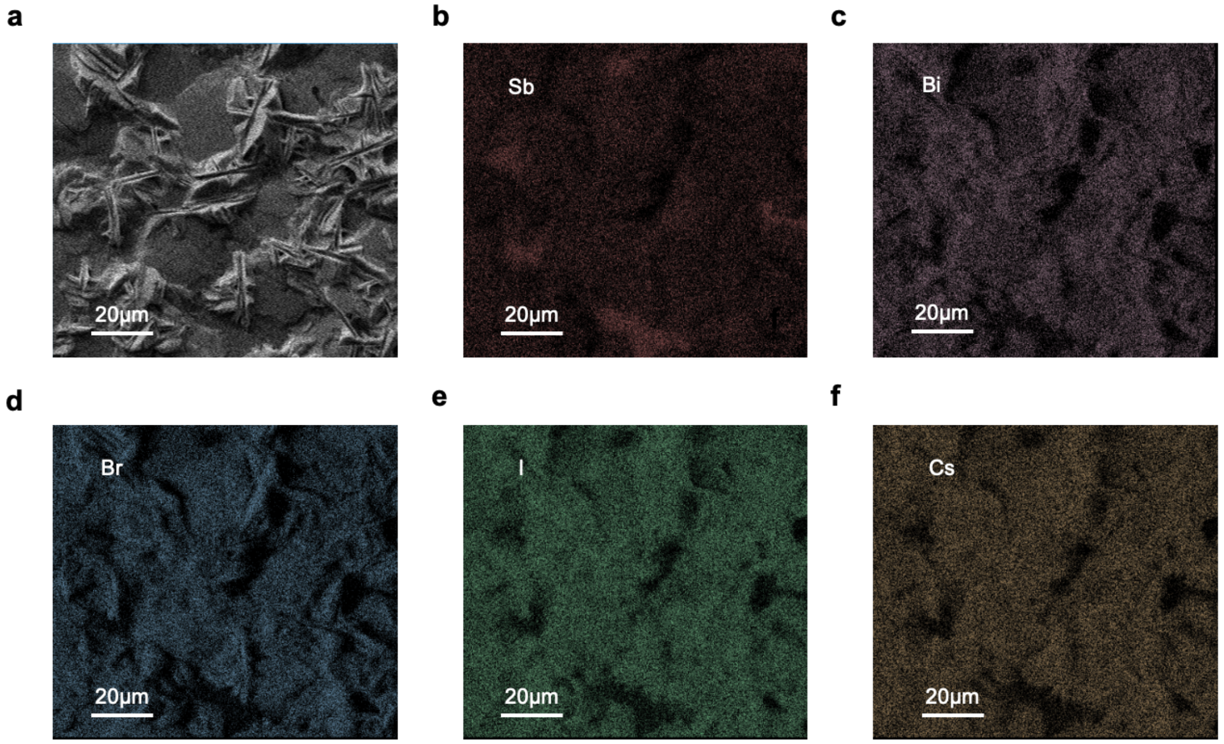

Supplementary Figure 11. SEM-EDS mapping on drop-casted sample with the composition $(Cs_3Sb_2Br_9)_{0.5}(Cs_3Bi_2I_9)_{0.5}$. a, SEM image of drop-casted $(Cs_3Sb_2Br_9)_{0.5}(Cs_3Bi_2I_9)_{0.5}$. **b-f,** EDS mapping of element **(b)** Sb**, (c)** Bi**, (d)** Br**, (e)** I and **(f)** Cs.

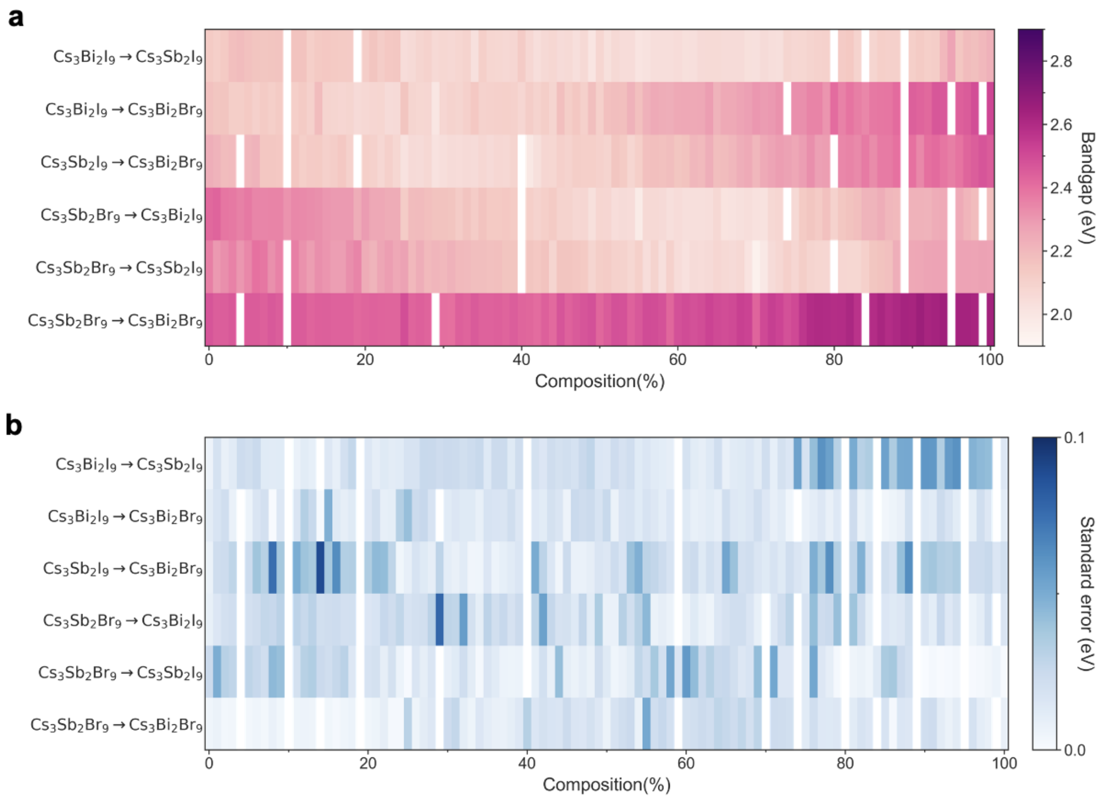

Supplementary Figure 12. Bandgap of six perovskite material gradients. a,**b**, (**a**) Average bandgaps and (**b**) standard errors calculated from the bulk reflectance spectra of six sequences based on three forward-backward gradient prints.

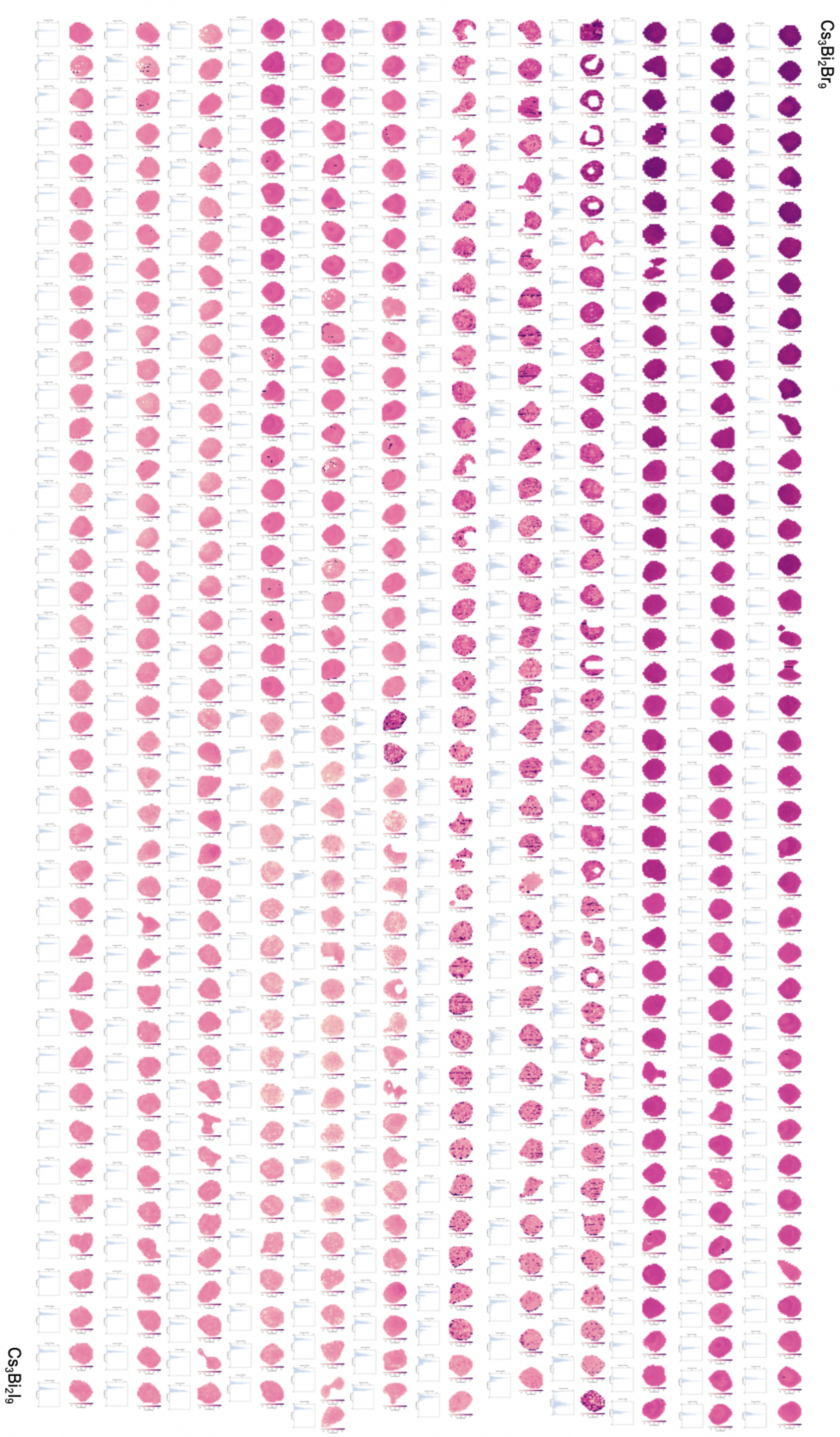

Supplementary Figure 13. Spatially resolved bandgap analysis results and histograms of all droplets in sequence $(Cs_3Br_2I_9)_{1-x}(Cs_3Bi_2Br_9)_x$ from 6 rounds of experiments.

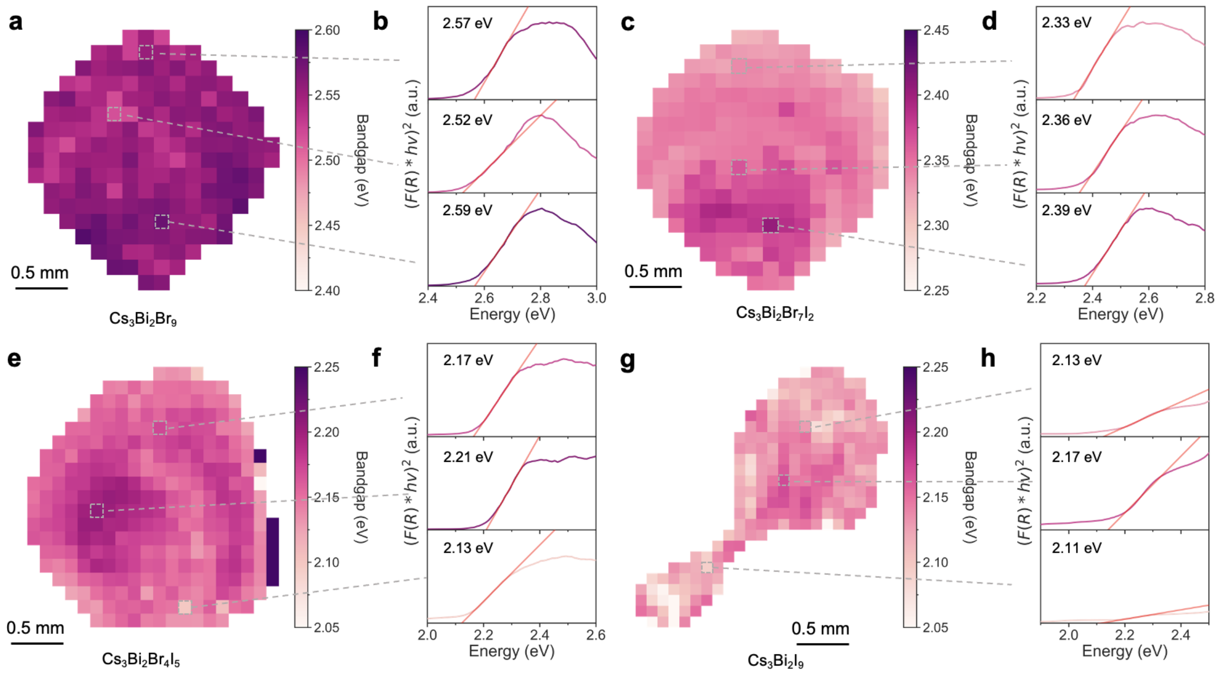

Supplementary Figure 14. Example local bandgap maps of droplets from the composition gradient sequence ($Cs_3Br_2I_9$)$_{1-x}$($Cs_3Bi_2Br_9$)$_x$. a,c,e,g, bandgap maps of droplets with compositions **(a)** $Cs_3Bi_2Br_9$, **(c)** $Cs_3Bi_2Br_7I_2$, **(e)** $Cs_3Bi_2Br_4I_5$, **(g)** $Cs_3Bi_2I_9$. **b,d,f,h,** pixel bandgap extraction results from the droplets with compositions **(b)** $Cs_3Bi_2Br_9$, **(d)** $Cs_3Bi_2Br_7I_2$, **(f)** $Cs_3Bi_2Br_4I_5$, **(h)** $Cs_3Bi_2I_9$. “a.u.” denotes “arbitrary units”.

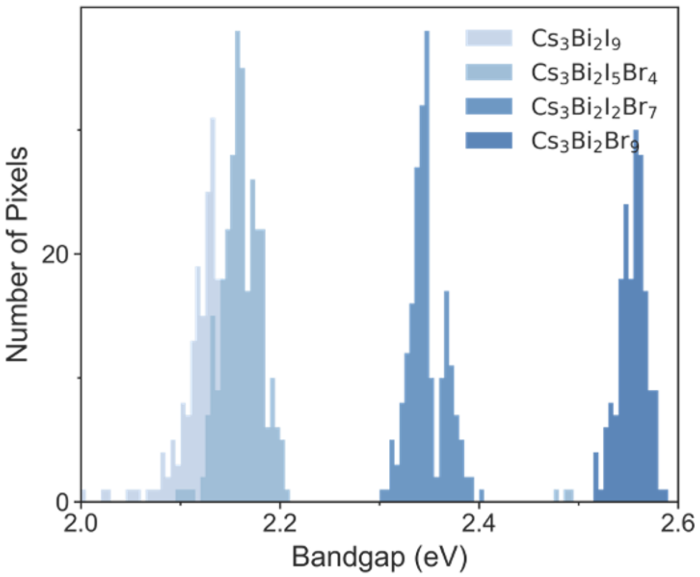

Supplementary Figure 15. Histograms of the bandgap distribution for droplets with different compositions.

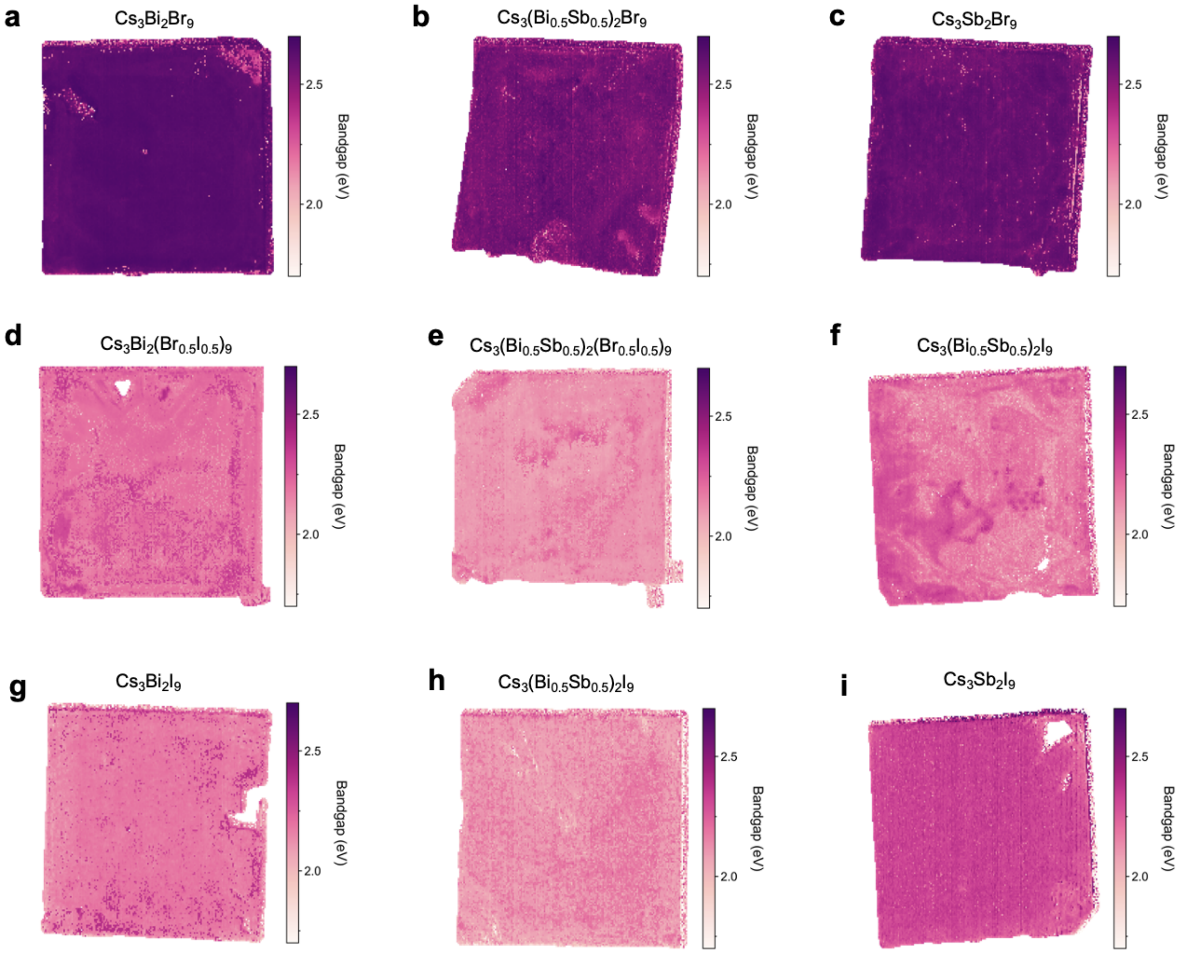

Supplementary Figure 16. Local bandgap maps of spin-coated thin films with compositions a, $Cs_3Bi_2Br_9$, **b,** $Cs_3(Bi_{0.5}Sb_{0.5})_2Br_9$, **c,** $Cs_3Sb_2Br_9$, **d,** $Cs_3Bi_2(Br_{0.5}I_{0.5})_9$, **e,** $Cs_3(Bi_{0.5}Sb_{0.5})_2(Br_{0.5}I_{0.5})_9$, **f,** $Cs_3(Bi_{0.5}Sb_{0.5})_2I_9$, **g,** $Cs_3Bi_2I_9$, **h,** $Cs_3(Bi_{0.5}Sb_{0.5})_2I_9$, **i,** $Cs_3Sb_2I_9$ **corresponding to batch-printed samples.**

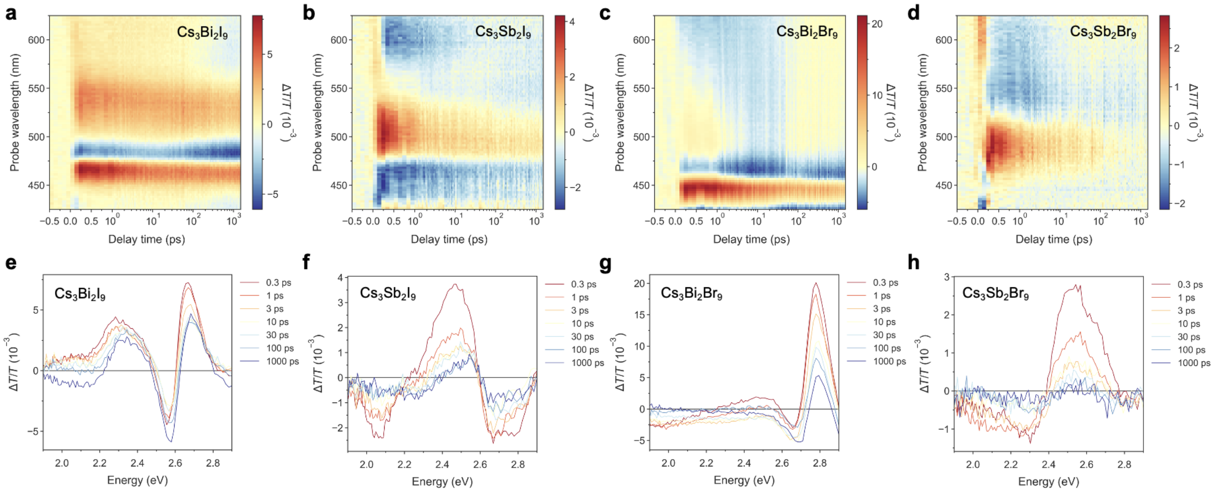

Supplementary Figure 17. Two-dimensional TA spectra and TA spectra at different time delays of four pure compositions. a,e, $Cs_3Bi_2I_9$. **b,f,** $Cs_3Sb_2I_9$. **c,g,** $Cs_3Bi_2Br_9$. **d,h** $Cs_3Sb_2Br_9$.

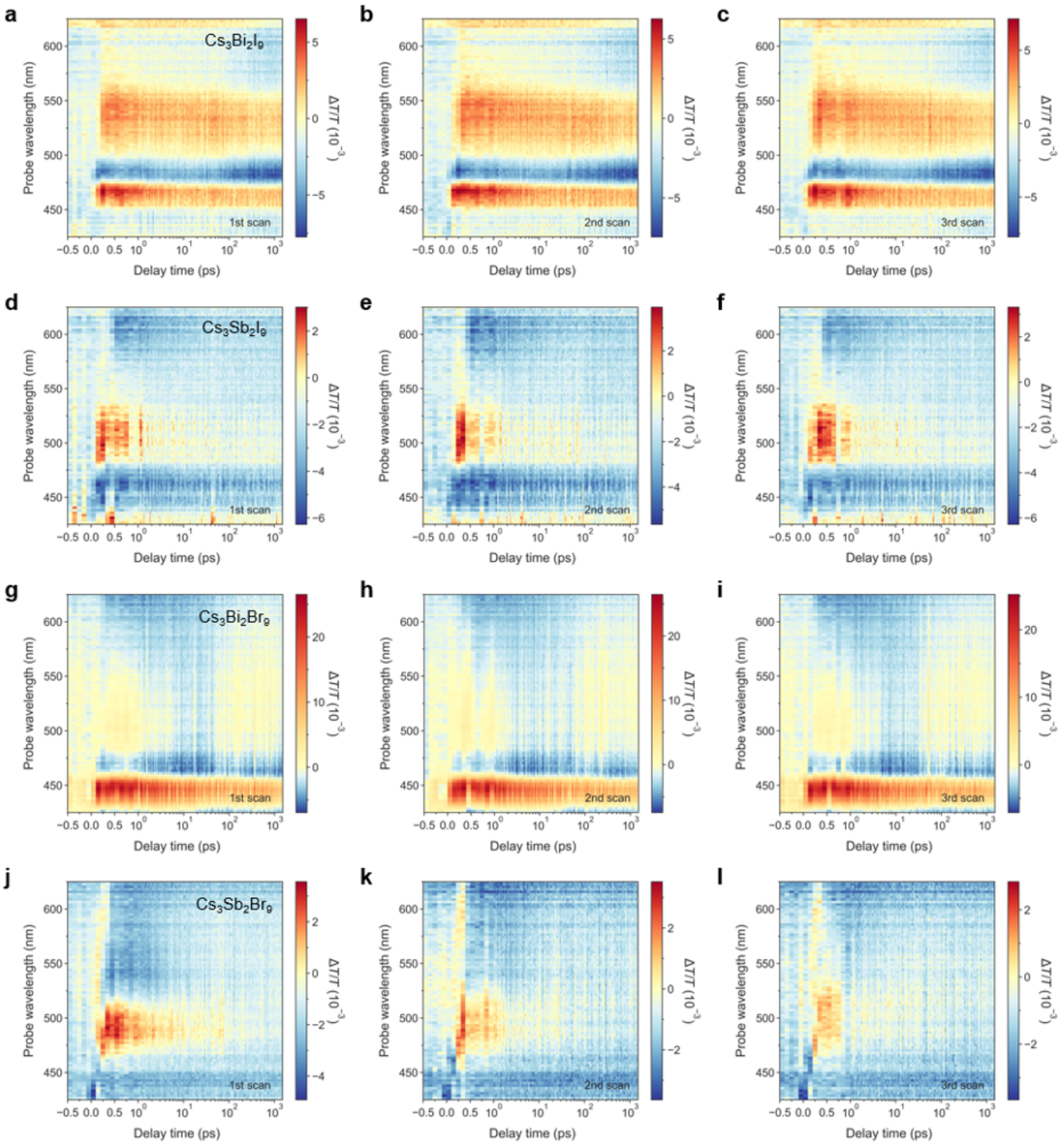

Supplementary Figure 18. TA spectra of four pure compositions in three rounds of scan. a-c, round 1-3 of composition $Cs_3Bi_2I_9$. **d-f,** round 1-3 of composition $Cs_3Sb_2I_9$. **g-i,** round 1-3 of composition $Cs_3Bi_2Br_9$. **j-l,** round 1-3 of composition $Cs_3Sb_2Br_9$.

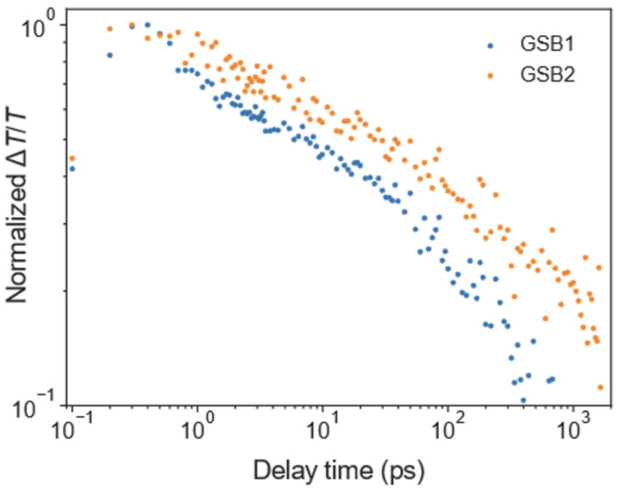

Supplementary Figure 19. Normalized transient absorption kinetics at two GSB peak positions in Figure 3d. GSB1: 2.28 eV; GSB2: 2.62 eV.

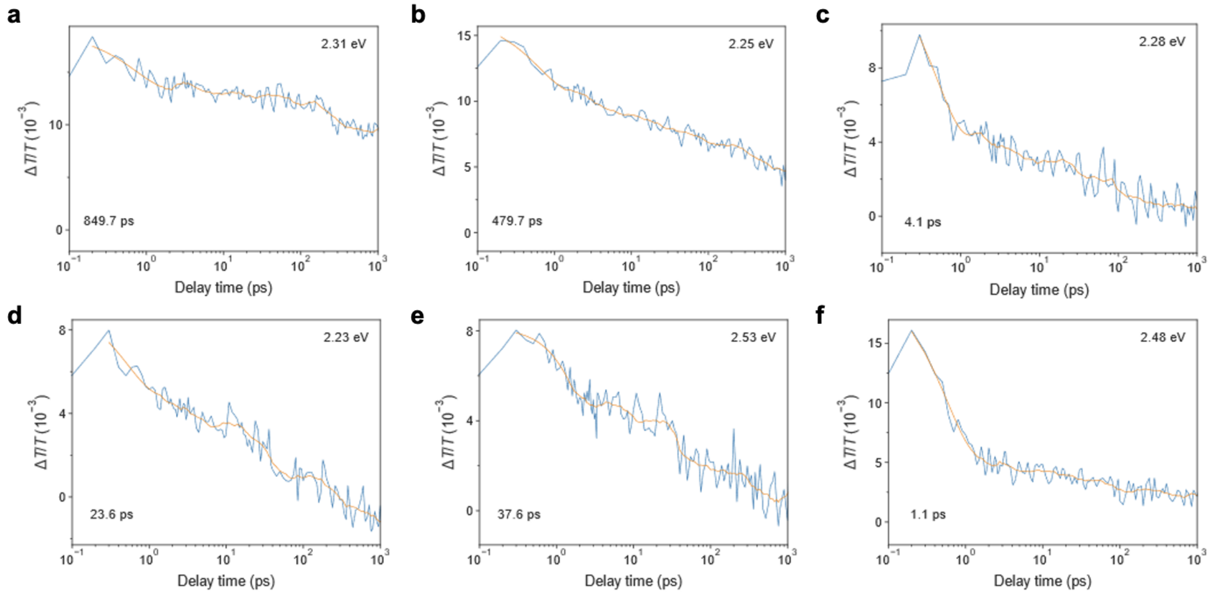

Supplementary Figure 20. Examples of lifetime fitting in transient absorption kinetics. The raw data (blue lines) was smoothed by applying a Savitzky-Golay filter with a window length of 51 and a polyorder of 3. The 1/e lifetime was then extracted from the smoothed curve (orange lines). **a**, $Cs_3Bi_2I_9$. **b**, $(Cs_3Bi_2I_9)_{0.2}(Cs_3Sb_2I_9)_{0.8}$. **c**, $(Cs_3Bi_2I_9)_{0.4}(Cs_3Sb_2I_9)_{0.6}$. **d**, $(Cs_3Bi_2I_9)_{0.6}(Cs_3Sb_2I_9)_{0.4}$. **e**, $(Cs_3Bi_2I_9)_{0.8}(Cs_3Sb_2I_9)_{0.2}$. **f**, $Cs_3Sb_2I_9$.

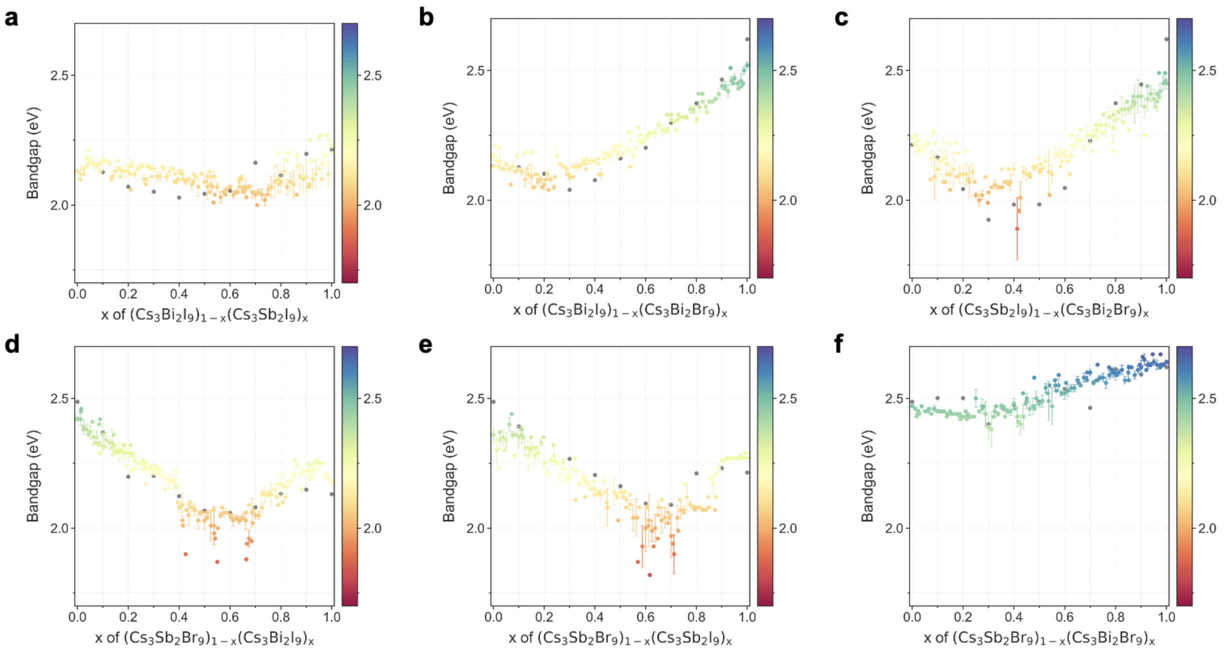

Supplementary Figure 21. Bandgap comparison between gradient-printed samples and spin-coated films. The grey dots represent bandgaps of spin-coated films. The colored dots represent average bandgaps of high-throughput samples from three rounds of experiments. The error bar represents the standard deviation of bandgaps in three rounds of high-throughput experiments. **a**, Sequence $(Cs_3Bi_2I_9)_{1-x}$ $(Cs_3Sb_2I_9)_x$. **b**, Sequence $(Cs_3Br_2I_9)_{1-x}$ $(Cs_3Bi_2Br_9)_x$. **c**, Sequence $(Cs_3Sb_2I_9)_{1-x}$ $(Cs_3Bi_2Br_9)_x$. **d**, Sequence $(Cs_3Sb_2Br_9)_{1-x}$ $(Cs_3Bi_2I_9)_x$. **e**, Sequence $(Cs_3Sb_2Br_9)_{1-x}$ $(Cs_3Sb_2I_9)_x$. **f**, Sequence $(Cs_3Sb_2Br_9)_{1-x}$ $(Cs_3Bi_2Br_9)_x$.

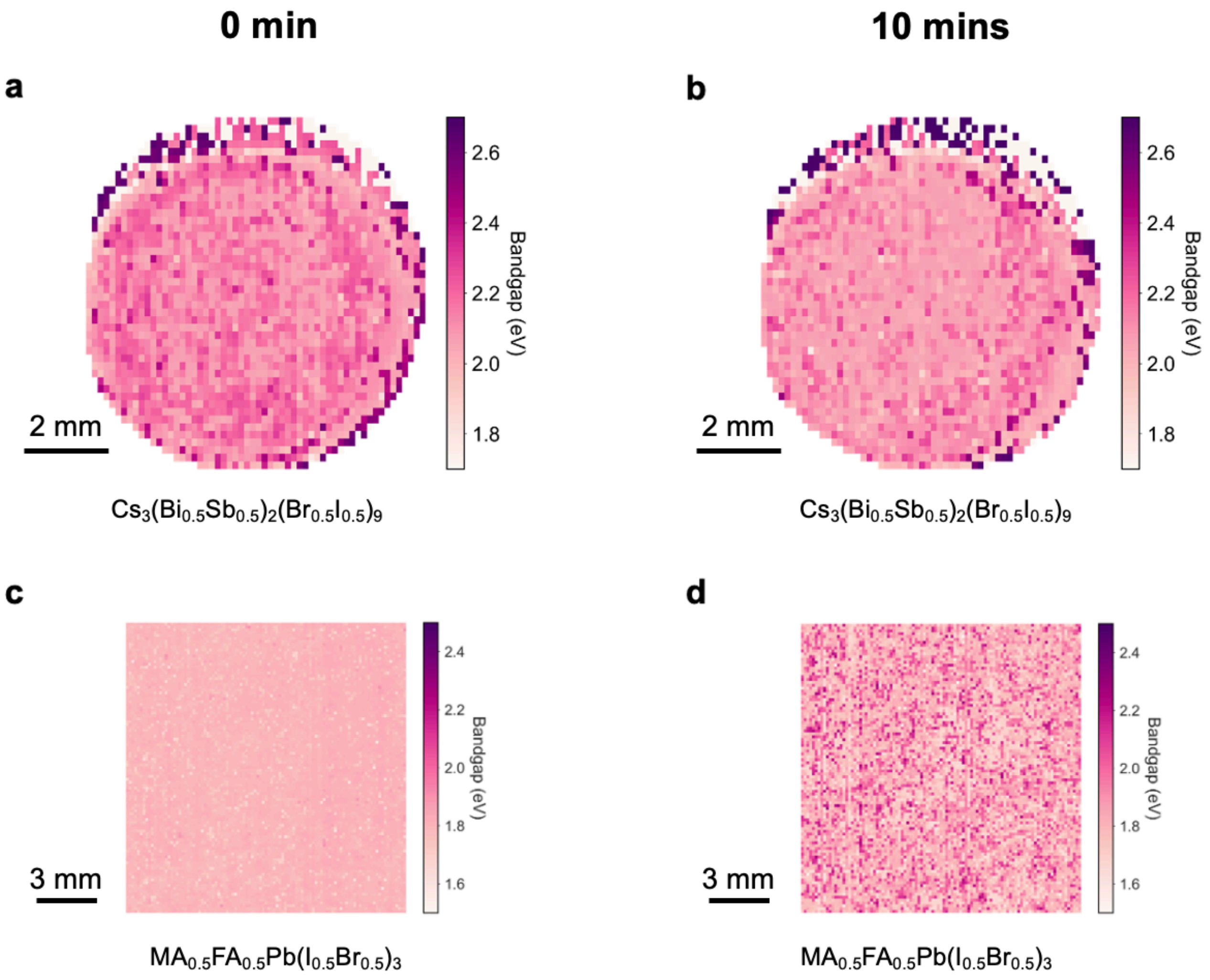

Supplementary Figure 22. Local bandgap maps of drop-casted $Cs_3(Bi_{0.5}Sb_{0.5})_2(Br_{0.5}I_{0.5})_9$ droplet and spin-coated $MA_{0.5}FA_{0.5}Pb(I_{0.5}Br_{0.5})_3$ film before and after 10-minute illumination. a and **b**, Bandgap maps of $Cs_3(Bi_{0.5}Sb_{0.5})_2(Br_{0.5}I_{0.5})_9$ droplet before and after 10-minute illumination under the light source of hyperspectral camera. **c** and **d,** Bandgap maps of $MA_{0.5}FA_{0.5}Pb(I_{0.5}Br_{0.5})_3$ film before and after 10-minute illumination under the light source of the hyperspectral camera.
